# Supplementary material for: Have environmental regulations promoted green technological innovation in cities? Evidence from China’s green patents
Source: PLoS One. 2022 Dec 13;17(12):e0278902. doi: 10.1371/journal.pone.0278902 (PMC9747053; doi:10.1371/journal.pone.0278902)
Supplement: Attachment — Submitted filename: PONE-D-22-16530.docx [file pone.0278902.s002.docx]

Studying the green development considerations is important and topical. Therefore, it is worth appreciating that the authors took care of it. Research on the relationship between environmental regulation and green technology innovation should be considered valuable and important. Nevertheless, authors need to correct a few important elements to make the article suitable for publication in a prestigious journal:

1. The range of literature sources are inadequate. Specifically, it didn’t include most recent literature related to enhance the validity of the research questions and related argument. Therefore, it has ignored most recent literature from the related field.

2. In section 3.1, the explanation of the mediation effect test steps should refer to relevant literature.

3. The impact of environmental regulation on green technology innovation will take some time to complete. How to test the dynamic effect or lagged effect?

4. In section 3.3, should explain the basis for data selection, that is, explain the reason for the deadline of data in 2019. If there are new policy regulations in the last two years, the research findings need to be discussed in relation to the latest policy.

5. This paper should create a discussion section that will answer the question of what the new article brings to science and how it contributes to the strengthening of green development. And the author should make comparison, contrast of previous paper and this paper to highlight the theoretical importance.

6. In conclusion, this paper should indicate the directions of further research and research limitations.
